# *BmSPP* is a virus resistance gene in *Bombyx mori*

**DOI:** 10.3389/fimmu.2024.1377270

**Published:** 2024-03-22

**Authors:** Yu-ting Feng, Chun-yan Yang, Lin Wu, Yuan-cheng Wang, Guan-wang Shen, Ping Lin

**Affiliations:** Integrative Science Center of Germplasm Creation in Western China (Chongqing) Science City, Biological Science Research Center, Southwest University, Chongqing, China

**Keywords:** antiviral, SPP, *Bombyx mori*, BmNPV, CRISPR/Cas9

## Abstract

**Introduction:**

Signal peptide peptidase (*SPP*) is an intramembrane protease involved in a variety of biological processes, it participates in the processing of signal peptides after the release of the nascent protein to regulate the endoplasmic reticulum associated degradation (ERAD) pathway, binds misfolded membrane proteins, and aids in their clearance process. Additionally, it regulates normal immune surveillance and assists in the processing of viral proteins. Although *SPP* is essential for many viral infections, its role in silkworms remains unclear. Studying its role in the silkworm, *Bombyx mori* , may be helpful in breeding virus-resistant silkworms.

**Methods:**

First, we performed RT-qPCR to analyze the expression pattern of *BmSPP*. Subsequently, we inhibited *BmSPP* using the *SPP* inhibitor 1,3-di-(N-carboxybenzoyl-L-leucyl-L-leucylaminopropanone ((Z-LL)_2_-ketone) and downregulated the expression of *BmSPP* using CRISPR/Cas9 gene editing. Furthermore, we assessed the impact of these interventions on the proliferation of *Bombyx mori* nucleopolyhedrovirus (BmNPV).

**Results:**

We observed a decreased in the expression of *BmSPP* during viral proliferation. It was found that higher concentration of the inhibitor resulted in greater inhibition of BmNPV proliferation. The down-regulation of *BmSPP* in both in vivo and in vitro was found to affect the proliferation of BmNPV. In comparison to wild type silkworm, *BmSPP^KO^* silkworms exhibited a 12.4% reduction in mortality rate.

**Discussion:**

Collectively, this work demonstrates that *BmSPP* plays a negative regulatory role in silkworm resistance to BmNPV infection and is involved in virus proliferation and replication processes. This finding suggests that *BmSPP* servers as a target gene for BmNPV virus resistance in silkworms and can be utilized in resistance breeding programs.

## 1 Introduction

*SPP* is a GXGD-type intramembrane-cleaving aspartyl protease with 9 transmembrane domains that can cleave transmembrane proteins in hydrophobic lipid bilayers (1, 2). *SPP* exhibits a highly level of conservation throughout evolution and is widely present in various eukaryotes, including fungi, protozoa, plants and animals (3). It possesses extensive biological functions: regulation of the ERAD pathway by eliminating signal peptides accumulated in the endoplasmic reticulum (ER) following cleavage by precursor signal peptidase (SP) (4); binding to misfolded membrane proteins and forming large oligomeric complexes that participate in autophagy *in vivo* (5); controlling normal immune surveillance by hydrolyzing signal peptides, promoting the release of epitope-containing fragments, and shielding cells from attack by Natural Killer cells (NK) (6); interacting with viral proteins, influencing viral processing and replication, or serving as a means for the virus to evade the host immune system (4, 7–9).

The organism’s own resistance to viruses has been greatly impacted by the knockdown or inhibition of *SPP*. *SPP* mediated cleavage is responsible for directing hepatitis C virus (HCV) core proteins to lipid droplets, a critical step for viral budding and nucleocapsid assembly. Inhibiting *SPP* with inhibitors has been shown to hinder HCV proliferation (7, 8, 10). During infection, herpes simplex virus (HSV) utilizes its glycoprotein K (gK) to bind to *SPP* to promote HSV-1 replication. The virus latency of *SPP*-induced knockout mice is significantly reduced, and the replication of the virus is also markedly reduced with *SPP* inhibitors (9, 11). *SPP* plays a role in the processing and maturation of classical swine fever virus (CSFV) core protein, and using (Z-LL)_2_-ketone to inhibit *SPP* can significantly diminish the viability of CSFV (12). These instances highlight the profound significance of *SPP* in viral infection, suggesting that targeting host *SPP* could be a highly effective antiviral strategy. The silkworm, *Bombyx mori*, is an economic insect due to its unique silk properties. However, the production of silkworms is often plagued by various sericultural diseases. Among these diseases, BmNPV is the most serious and costly viral disease, resulting in significant sericultural losses. Considering the properties of *SPP*, we investigated whether editing *BmSPP* could improve the resistance of silkworms to BmNPV. Our expectation was that editing of *BmSPP* would yield a resistant strain.

NPV is a baculovirus that exists in a variety of arthropods and can infect over 600 species of insects in eight orders, including *Lepidoptera*, *Hymenoptera*, *Diptera*, *Coleoptera*, etc (13). It is a DNA virus with a double-chained circular DNA genome and gets its name for its genome being encased in a rod-shaped nuclear capsid (14). BmNPV produces two types of virus particles during infection: the occlusion-derived virus (ODV) and the budded virus (BV). Baculovirus infection of the host larva is caused by ODV, and subsequently, BV causes systemic infection in the host (15). Baculovirus enters the host through the mouth, travel through the foregut and enter the midgut, and releases ODVs in the alkaline environment of the midgut. Then ODVs fuse directly with midgut cell membranes and release the nucleocapsid into the cytoplasm, leading to primary infection (14). In the host, the virus takes advantage of the host’s own environment to replicate in the nucleus and assemble the nucleocapsid to produce budding virions, BVs. These BVs infect other tissues such as fatbody and muscle with the help of the host’s trachea or hemocytes, resulting in systemic infection, also known as secondary infection (16, 17). In the later stage of viral infection, a large number of new viral particles, ODVs, are produced. The ODVs become embedded in the nucleus of the polyhedron protein to form an occlusion body (OB), which ultimately leads to cloudy blood, cuticle rupture, and the extravasation of white pus, causing morbidity and death (13).

In this study, we first inhibited *BmSPP* in BmN cells by using (Z-LL)_2_-ketone, which is a commonly used inhibitor of *SPP*. As a result, we observed a significant decrease in BmNPV proliferation. Additionally, we achieved the same inhibitory effect on BmNPV proliferation by knocking down *BmSPP* in the cells through the use of CRISPR/Cas9 gene editing technique. To successfully knocked down *BmSPP*, we utilized transgenic microinjection into embryos of a non-lagging *Bombyx mori* strain, Dazao, coupled with CRISPR/Cas9 gene editing. The *BmSPP^KO^* silkworm displayed a significantly increased resistance to BmNPV compared to *BmSPP^WT^*. Importantly, these genetic modifications did not negatively impact the economic traits of silkworms. Taken together, our findings suggest that *BmSPP* as a potential candidate gene for enhancing BmNPV resistance. By leveraging this knowledge, we can improve the resistance of silkworms against viral infection, ultimately minimizing the losses incurred during rearing due to BmNPV infection.

## 2 Materials and methods

### 2.1 Bioinformatics analysis

Amino acid sequences of *BmSPP* and its homologs in other insects of different orders as well as in various model organisms were retrieved from the NCBI database (https://www.ncbi.nlm.nih.gov/). These species include *Bombyx mandarina*, *Manduca sexta*, *Cryptotermes secundus*, *Coptotermes formosanus, Ctenocephalides felis*, *Athalia rosae*, *Lamprigera yunnana*, *Nilaparvata lugens*, *Musca domestica*, *Daphnia pulex*, *Drosophila arizonae*, *Homo sapiens*, *Mus musculus*, *Danio rerio* and *Xenopus laevis.* For Multiple sequence comparison and coloring, we utilized Jalview software. To compare sequences, we imported *SPP* sequences and selected “Web Service-Alignment-ClustalO-with Defaults” and to add a color, we selected “Colour-Clustal”. Further, we predicted the conserved motif using the MEME (https://meme-suite.org/meme/tools/meme). After importing the sequence, we adjusted the “Select number of patterns” option to 10 and click “Search”.

### 2.2 Cell line, silkworm strain and viruses

The *Bombyx mori* strain Dazao (DZ) and BmN cell line were maintained at the Integrative Science Center of Germplasm Creation in Western China (Chongqing) Science City, Biological Science Research Center (Southwest University, Chongqing, 400716, China). BmN cells were cultured at 26 °C in Grace medium supplemented with 10% fetal bovine serum (FBS; Gibco) and penicillin and streptomycin (18). The rearing temperature of silkworm larvae was 26 ± 1 °C, and the larvae fed on fresh mulberry leaves. BmNPV BVs expressing green fluorescent protein (BmNPV-GFP) were collected from the infected BmN cells (19). BmNPV (Guangdong strain) was collected from the hemolymph of infected silkworm larvae.

### 2.3 RT-qPCR analysis of *BmSPP* expression

The eggs, L1D2, L2D0, L2D2, L3D0, L3D2, L4D0, L4D2, L5D1, L5D3, L5D5, L5D7 and pupa of DZ silkworms were used for RNA extraction. Total RNA of testis, ovary, malpighian tubule, hemolymph, fatbody, anterior silk gland, middle silk gland, posterior silk gland, head, epidermis, midgut and trachea of DZ silkworms were extracted at day-3 fifth instar larvae. RNA was extracted from the fatbody of L4D3, L5D1, L5D3, L5D5, L5D7 and from the midgut of L4D1, L4D3, L5D1, L5D3, L5D5, L5D7 of DZ silkworms. BmN cells were treated with BmNPV-GFP and collected at 3, 6, 12, 24, 48, and 72 hours post infection (hpi) for RNA extraction. Total RNA was extracted with TRIzol reagent (Invitrogen) and cDNA was synthesized with M-MLV reverse transcriptase (Promega). Translation initiation factor 4a (*TIF-4A*) is considered a housekeeping gene in silkworms (20), as its expression remains unaffected by experiments, making it commonly used in gene expression analysis. The cDNA from these samples was used for qPCR analysis of *BmSPP*, while control *TIF-4A* was used as a reference. Primer sequences were: *BmSPP* (F: GCTCTGTCTTGGAGCTTGGT, R: GTCCGCAGAGTAGGATGCAG) and *TIF-4A* (F: TTCGTACTGGCTCTTCTCGT, R: CAAAGTTGATAGCAATTCCCT). BmN cells were treated with BmNPV-GFP and collected at 3, 6, 12, 24, 48, and 72 hpi for total DNA extraction. Total DNA was extracted post-infection with a tissue DNA kit (Omega). Additionally, we using glyceraldehyde-3-phosphate dehydrogenase (*GAPDH*) as an internal reference gene, since its expression is not influenced by BmNPV attack, qPCR was performed to investigate the changes in the expression of BmNPV genes, *GP64*. Primer sequences were: *GAPDH* (F: CATTCCGCGTCCCTGTTGCTAAT, R: GCTGCCTCCTTGACCTTTTGC) and *GP64* (F: CCATCGTGGAGACGGACTA, R: CTCGCACTGCTGCCTGA). Fluorescence real time PCR analysis was performed using the SYBR Premix Ex Taq II (Takara) on a, 7500 rapid real-time PCR system (Applied Biosystems) at 95 °C for 30 s, 40 cycles at 95 °C for 5 s and 60 °C for 30 s.

### 2.4 Plasmid construction

The exon sequence of *BmSPP* was selected as the target region for gene knockdown. The sgRNA primers were designed on first exon using CCTop (https://cctop.cos.uni-heidelberg.de:8043/), and the sgRNA primer sequence with the highest score was chosen: *SPP*-sgRNA (F: AAGTGATGTTTATAGGTATTTCTG, R: AAACCAGAAATACCTATAAACATC). The synthesized sequence was annealed to produce double-stranded gRNA, which was then ligated to pBac[3×P3-EGFP-SV40-U6-TTTTTT] base vector (provided by the laboratory) by T4 ligase (NEB) (19). The ligated plasmid was transfected into competent cells, cultured overnight, and a single colony was picked for PCR verification. PrimeSTAR^®^ Max DNA polymerase (Takara) was used for PCR with the following procedure: 98 °C 2 min, 40 cycles at 98 °C for 10 s, 58 °C for 15 s, 72 °C for 30 s, and 72 °C for 5 min. These positive clones were the sequenced. Primer sequences were: U6R (AGCTGTCCAAGGAATGCG) and gRNA-F (CGACTCGGTGCCACTTT). The pBac[3×P3-EGFP-SV40-U6-gRNA-TTTTTT] knockout vector was constructed and named *SPP*-sgRNA. In this vector, 3×P3 represents an eye-specific expression promoter, EGFP refers to green fluorescent protein, SV40 denotes the termination sequence, U6 indicates the promoter, and TTTTTT represents the termination sequence.

### 2.5 Inhibitor treatment

The *SPP* inhibitor ((Z-LL)_2_-ketone) (Sigma) was dissolved in DMSO. Due to the toxicity of DMSO to cells, the concentration of DMSO in all experimental groups was maintained at 2.5%, a concentration that did not affect the morphology and viability. (Z-LL)_2_-ketone mixed with cell medium until the final concentration was 10 μM, 20 μM, 50 μM, 100 μM, and 200 μM, and added to BmN cells. In the control group, the medium was free of inhibitors and contained only 2.5% DMSO. 72 hours later cell morphology was observed by microscopy and 10% Cell Counting Kit-8 (Beyotime) was added and cell viability was assayed according to the manufacturer’s instructions. The appropriate concentration was selected for subsequent experiments, and after 6 h of inhibitor incubation, BmNPV-GFP was added. To analyze the proliferation of BmNPV-GFP, inverted fluorescence microscopy (Leica, DMi8) was performed after cells were infected with viruses at 0, 12, 24, and 48 hpi, and then cells were collected for total DNA extraction. Tissue DNA Kit (Omega) was used to extract total DNA. *GAPDH* was used as an internal control to detect DNA abundance of BmNPV through the expression of *IE1*, *Helicase*, *GP64* and *VP39*. Primer sequences were: *GAPDH* (F: CATTCCGCGTCCCTGTTGCTAAT, R: GCTGCCTCCTTGACCTTTTGC); *IE1* (F: CACGGTCGCTTCAACTCAA, R: TGTCGTCGAAACGCATCAA); *Helicase* (F: AACACATGCCAAGCCGATAT, R: TCCCGACACCGTTGACC); *GP64* (F: CCATCGTGGAGACGGACTA, R: CTCGCACTGCTGCCTGA) and *VP39* (F: TAATGCCCGTGGGTATGG, R: GTTTGATGAGGTGGCTGTTGC).

### 2.6 *BmSPP* knockout in BmN cells

The *nos*-Cas9 vector expresses Cas9 protein throughout the growth process and tissues of silkworms (21). Transgenic knockout vector *SPP*-sgRNA was mixed with *nos*-Cas9 plasmid at 1:1 molar ratio to obtain the most effective gene knockout. X-tremeGENE HP DNA transfection reagent (Roche) was used to transfect BmN cells, with the transfection reagent serving the control. 48 hours after transfection, the exogenously introduced DNA integrated better into the genome of the cells as the cells themselves proliferated. Total DNA was extracted at 48 h, and genomic PCR was performed with PrimeSTAR ^®^Max DNA polymerase (Takara). The procedure was as follows: 98 °C 2 min, 40 cycles at 98 °C for 10 s, 54 °C for 15 s, 72 °C for 30 s, and 72 °C for 5 min, then the product was ligated with pEASY^®^-Blunt Zero Cloning Kit (TransGen), and after transformation, monoclonals were selected for sequencing to detect whether knock-out occurs. Primer sequences were: *SPP-*KO (F: ACTATCCGTAGGGGAAAGTTGTC, R: GAAACGATTCGATGCATTGA). The transfected BmN cells were infected with BmNPV-GFP after cultured 48 h. Total DNA was extracted at 0, 12, 24, 48, and 72 hpi. Tissue DNA Kit (Omega) was used to extract total DNA after infection for qPCR analysis. *GAPDH* was used as an internal control to detect DNA abundance of BmNPV through the expression of *IE1*, *Helicase*, *GP64* and *VP39*.

### 2.7 Generation and mortality analysis of transgenic silkworms

The transgenic knockout vector *SPP*-sgRNA was mixed with an equal amount of A3 helper plasmid (provided by our laboratory) at 1:1 molar ratio and microinjected into fresh embryos within 2 h of egg laying of the silkworms. Surviving G0 moths were self-crossed and generate G1 broods. EGFP-labeled positive transgenic individuals were screened by fluorescence microscopy (Olympus), which named *BmSPP*-gRNA. *BmSPP*-gRNA was then crossed to *Hsp90*-Cas9 strain that expresses Cas9 protein in the whole body and red fluorescent protein RFP in the eyes (provided by our laboratory). Knockout individuals with both EGFP and RFP markers in the eyes were screened under fluorescence microscopy, and then fifth instar knockout larvae were selected to extract whole silkworm genomic DNA for knockout detection. Day-0 fifth instar larvae of transgenic silkworms *BmSPP^KO^* and wild type silkworms *BmSPP^WT^* were orally infected with BmNPV using 2×10^6^ OB/larvae. Each infected line test consisted of four replicates and each repeat included 40 larvae. Three larvae from the fourth repetition were collected as a mixed sample at 1, 2, 3, 4, 5, and 6 days post infection. Total DNA was extracted and analyzed by qPCR for the BmNPV gene, *GP64* and control *GAPDH* to detect changes in viral DNA and calculated the mortality rate of the first three replicates.

### 2.8 Phenotypic observation

The transgenic silkworms *BmSPP^KO^* and wild type silkworms *BmSPP^WT^* were reared until pupation, and male and female identification was carried out on the pupae of 5-day-old, and the full cocoon weight, cocoon layer weight and cocoon layer rate of males and females were investigated in groups.

### 2.9 Statistical analysis

Statistical analysis was performed with GraphPad (GraphPad Software, LaJolla, CA) using student’s t-tests. No significant difference between samples is indicated as P>0.05 and statistically significant differences are indicated as ns P>0.05, * P<0.05, ** P<0.01, *** P<0.001, **** P<0.0001.

## 3 Results

### 3.1 Expression patterns of *BmSPP*

Bioinformatics analyses showed high homology and conservation of *SPP* across species, and MEME analyses indicated the presence of two conserved motifs in these species (**Supplementary Figure S1**). It is hypothesized that *SPP* may be involved in the same biological functions in different species.

**Figure 1:**
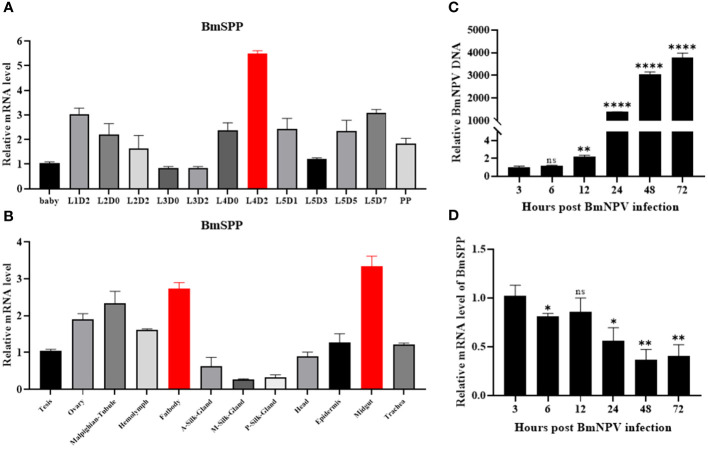
Expression analysis of *BmSPP.* **(A)** qPCR detection of *BmSPP* expression at different stages during the growth of silkworm strains. **(B)** qPCR was used to detect *BmSPP* expression in different tissues of day-3 fifth instar larvae. **(C)** Viral proliferation curve. BmNPV-GFP infected BmN cells and total DNA was extracted at 3, 6, 12, 24, 48 and 72 hpi. The accumulated viral DNA content was detected by qPCR of the BmNPV gene, *GP64*. **(D)** Changes of *BmSPP* expression in BmN cells after infection. Data are given as mean ± SD (n = 3). Student’s t-tests were used for statistical analysis (ns P>0.05, * P<0.05, ** P<0.01, **** P<0.0001).

The RT-qPCR was used to analyze the expression pattern of *BmSPP* at different growth stages of *Bombyx mori* and in different tissues of day-3 fifth instar larvae. For qPCR analysis, cDNA from different growth stages of silkworm were utilized and revealed that *BmSPP* was expressed in all stages, with the highest expression level observed on day-2 fourth instar larvae (**Figure 1A**). Additionally, *BmSPP* was expressed in all tissues of day-3 fifth instar larvae, with a higher expression level in midgut and fatbody, which are the main immune organs of silkworms (**Figure 1B**). Subsequently, the expression of *BmSPP* was detected in the midgut and fatbody at different growth periods, and it was found to be expressed at all periods (**Supplementary Figure S2**). We hypothesized that *BmSPP* might play a role in antiviral immunity. BmN cells were infected with BmNPV-GFP and the proliferation curve of the virus was plotted (**Figure 1C**) and the changes in the expression level of *BmSPP* at different periods of viral infection were detected. The qPCR results showed that a decrease in the expression level of *BmSPP* after viral infection (**Figure 1D**).

### 3.2 Inhibition of *BmSPP* by (Z-LL)_2_-ketone affects BmNPV proliferation

**Figure 2:**
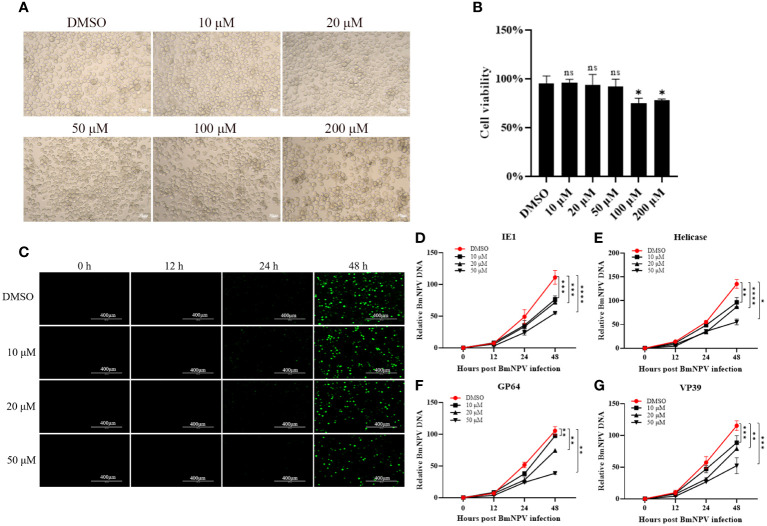
BmNPV proliferation was inhibited after treatment of *BmSPP* inhibitor (Z-LL)_2_-Ketone. **(A)** The cell growth of BmN cells after 72 hours of incubation with (Z-LL)_2_-ketone was observed under a microscope. **(B)** Cell viability assay after 72 hours of drug administration. **(C)** After incubation with different concentrations of inhibitor, BmN cell was infected by BmNPV-GFP and observed the viral fluorescence at 0, 12, 24, and 48 hpi. **(D-G)** After incubation with different concentrations of inhibitors, the expression of BmNPV genes were determined by qPCR at 0, 12, 24, and 48 hpi at different infection stages, *IE1* **(D)**, *Helicase* **(E)**, *GP64* **(F)**, *VP39* **(G)**. Data are given as mean ± SD (n = 3). Student’s t-tests were used for statistical analysis (ns P>0.05, * P<0.05, ** P<0.01, *** P<0.001, **** P<0.0001).

There are many commercially available *SPP* inhibitors, the most widely used being (Z-LL)_2_-ketone, a transition state analog that mimics the leucine-rich hydrophobic amino acid sequences found in many *SPP/SPPL* substrates, thereby inhibiting substrate processing (11, 22). (Z-LL)_2_-ketone has not been documented for use in silkworm, we performed inhibitor toxicity assays after incubating BmN cells with different concentrations of inhibitors. The results showed that low concentrations of inhibitors (10, 20 and 50 μM) did not affect the normal cell morphology and were not toxic to the cells (**Figures 2A, B**).

BmN cells were treated with 10 μM, 20 μM, and 50 μM concentrations of the *SPP* inhibitor (Z-LL)_2_-ketone. These cells were then infected with BmNPV-GFP labeled with green fluorescent protein. Virus proliferation was observed at 0, 12, 24, and 48 hpi using fluorescence microscopy. Cells treated with the inhibitor showed significantly reduced virus fluorescence (**Figure 2C**). The expression of BmNPV genes can be categorized into four phases: very early (0-4 hpi), late early (5-7 hpi), late (8-18 hpi), and very late (>18 hpi) (23). To analyze virus proliferation after inhibitor treatment, genomic DNA was extracted from the samples and the expression of key viral proliferation genes such as *IE1* (**Figure 2D**), *Helicase* (**Figure 2E**), *GP64* (**Figure 2F**), and *VP39* (**Figure 2G**) was determined at different stages. The results showed that BmNPV proliferation was inhibited after inhibitor administration, with the degree of inhibitor becoming more prominent and higher the concentrations.

### 3.3 Knockdown of *BmSPP* protected BmN cells against BmNPV infection

**Figure 3:**
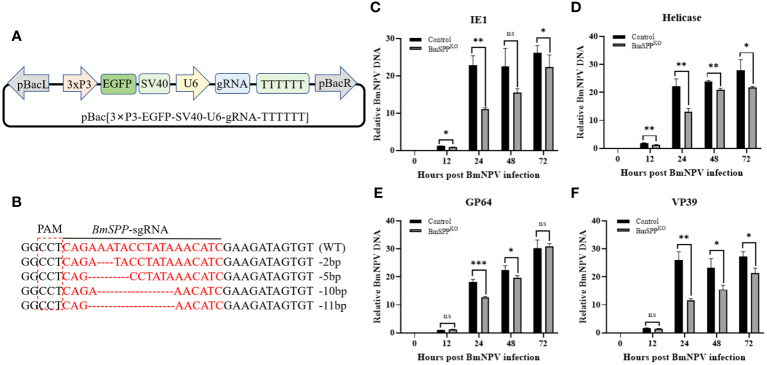
Inhibition of BmNPV proliferation in BmN cells was observed following the knockout of *BmSPP*. **(A)** Schematic diagram of the *SPP*-sgRNA expression vector. **(B)** Mutation of *BmSPP* in BmN cells. **(C–F)** Transfected cells were infected with BmNPV-GFP, and the BmNPV genes expression were determined by qPCR at 0, 12, 24, 48, and 72 hpi at different infection stages, *IE1* **(C)**, *Helicase* **(D)**, *GP64* **(E)**, *VP39* **(F)**. Data are given as mean ± SD (n = 3). Student’s t-tests were used for statistical analysis (ns P>0.05, * P<0.05, ** P<0.01, *** P<0.001).

Since the inhibition of *BmSPP* in BmN cells results in the inhibition of BmNPV proliferation, we aimed to determine whether *BmSPP* has the same inhibitory effect on BmNPV proliferation at the cellular level when *BmSPP* is knocked out. To achieve this, we constructed a *SPP-*sgRNA knockout vector for the silkworm (**Figure 3A**). After transfection, genome sequencing was performed at 48 hours, which confirmed the successful mutation of the *BmSPP* gene (**Figure 3B**). Subsequently, transfected BmN cells were infected with BmNPV-GFP. Total DNA was extracted at 0, 12, 24, 48 and 72 hpi and qPCR analysis revealed that the down-regulation of *IE1* (**Figure 3C**), *Helicase* (**Figure 3D**), *GP64* (**Figure 3E**), and *VP39* (**Figure 3F**) expression. These results suggest that the reduced expression of *BmSPP* can inhibit the proliferation of BmNPV.

### 3.4 BmNPV DNA content was decreased in *BmSPP^KO^* silkworm and does not affect the economic value

**Figure 4:**
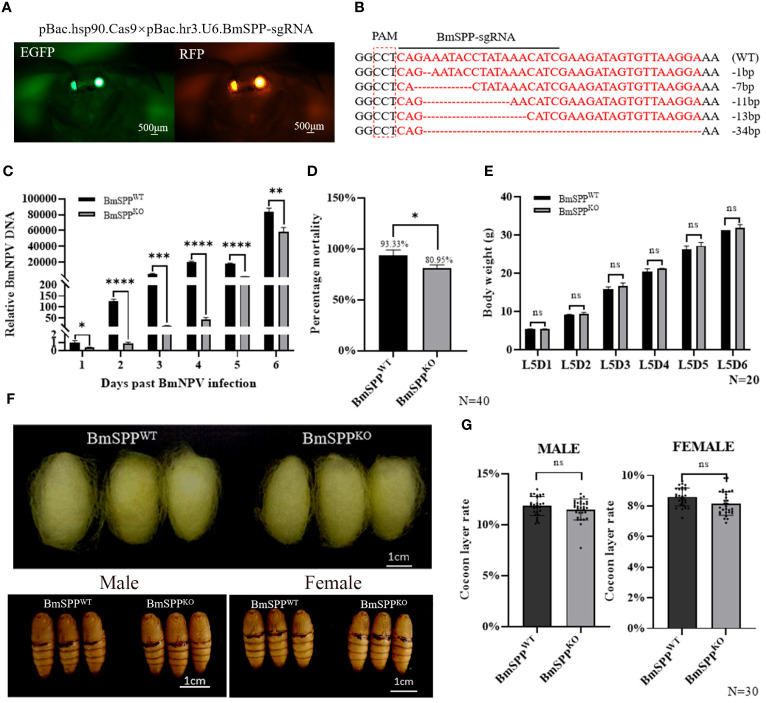
Antiviral capacity of *BmSPP^KO^* silkworm. **(A)** *BmSPP^KO^* strain screening method. **(B)** Part of the mutant Sequences. **(C)** Detection of viral DNA content. The BmNPV gene, *GP64* expression was determined by qPCR at 1, 2, 3, 4, 5, and 6 days post infection. **(D)** Mortality was recorded until to pre-pupation (n=40). **(E)** Body weight of *BmSPP^KO^* and *BmSPP^WT^*. **(F)** Phenotypic observation. **(G)** Cocoon layer rate of *BmSPP^KO^* and *BmSPP^WT^*. Data are given as mean ± SD (n = 3). Student’s t-tests were used for statistical analysis (ns P>0.05, * P<0.05, ** P<0.01, *** P<0.001, **** P<0.0001).

We utilized CRISPR/Cas9 gene editing technology to knock out *BmSPP* in *Bombyx mori.* First, a gRNA positive individual expressing the green fluorescence protein (EGFP) in the eyes was obtained through microinjection. Subsequently, it was crossed with *Hsp90*-Cas9 strains that express the Cas9 protein at the end instar larvae of the silkworm (24), and individuals exhibiting both green and red fluorescence markers in the eyes were selected (**Figure 4A**). Analysis of genome sequencing demonstrated the mutation of *BmSPP* (**Figure 4B**). Individual challenge experiments showed that the BmNPV DNA content in *BmSPP^KO^* was significantly lower than that in *BmSPP^WT^* (**Figure 4C**). Finally, the mortality rate from BmNPV infection to pre-pupation was assessed, showing that *BmSPP^KO^* had a mortality rate 12.4% lower than *BmSPP^WT^* (**Figure 4D**). In conclusion, the reduction in *BmSPP* level enhances the disease resistance of silkworm larvae to BmNPV, indicating its negative regulatory role in silkworm resistance to BmNPV infection.

When *BmSPP^WT^* and *BmSPP^KO^* were fed until the fifth instar stage, there were no significant changes in body weight between *BmSPP^WT^* and *BmSPP^KO^* (**Figure 4E**). Furthermore, we observed pupal phenotypes and determined that there were no significant differences in cocoon size and pupal size in the *BmSPP^KO^* compared to *BmSPP^WT^* (**Figure 4F**). Additionally, we also assessed the economic value of females and males, and likewise found no significant changes (**Figure 4G**). These results indicate that the knockout of *BmSPP*, facilitated by the *Hsp90*-Cas9 strain, does not impact the economic value of the silkworm. This finding is critical for the development of NPV-resistant breeding.

## 4 Discussion

BmNPV is the most common and serious disease of the silkworm. BmNPV DNA replication initiates at 8 hours post infection (25). Based on the aforementioned results, it is evident that BmNPV DNA content undergoes significantly changes compared to the control group after 12 h of infection, regardless of *SPP* inhibitor medication or *BmSPP* knockout in the cells. Therefore, the antiviral effect of *BmSPP* may be related to the replication of viral DNA, and the results of the virus tapping assay on the knockout individuals also showed that it inhibited the proliferation of the virus. The knockout of *BmSPP* caused by *Hsp90*-Cas9 did not cause any abnormalities in the development of individuals, and the morphology and size of the cocoons did not change, nor did the rate of cocoon layer change. The results revealed that *BmSPP* exerts a negative regulatory role in silkworm resistance to BmNPV infection.

*SPP* possesses a wide range of biological functions, exerting its influence on multiple central cellular pathways primarily by controlling the levels of membrane substrate proteins (26). This implies its significant implications for physiological functions and diseases. The current study focuses primarily on remarkable antiviral and anti-plasmodium infection capabilities of *SPP* (4). As previously mentioned, *SPP* negatively regulates various viral infections through different mechanisms of action and is a very important immune-related target gene. To our knowledge, there are no studies have reported that changes in *SPP* in silkworms can have similar regulatory effects on viruses of silkworms. In this study, we discovered for the first time that *BmSPP* contributes to the DNA replication of the virus and impacts the proliferation of BmNPV, suggesting that *BmSPP* could be a potential target for BmNPV prevention. However, further experimental exploration is required to comprehend the specific mechanism of action.

*Bombyx mori* holds significant economic value. With the advancement of artificial feed and intelligent breeding, the future trends of silkworm breeding will be characterized by large-scale, multi-batch and high-density breeding. However, the thorough disinfection of sericulture sites and equipment remains a challenge, leading to the spread and accumulation of pathogens. Furthermore, most disinfection techniques can only partially reduce pathogens in the environment, and the current silkworm varieties often exhibit low resistance to pathogens. Consequently, it becomes easier to induce silkworm disease and incur substantial loss, leading to frequent outbreaks of BmNPV infection. Sericulturists have long anticipated the development of silkworm varieties resistant to high levels of NPV in order to confront the threat of silkworm disease. Traditional breeding methods for BmNPV resistant varieties typically involve selecting silkworm species with exceptional breeding performance as foundation. By exposing individuals to high concentration of BmNPV, survivors can be used for subsequent breeding, allowing for simultaneous selection of economic traits and the enhancement of disease resistance through multiple virus additions during subculturing. Modern methods for enhancing resistance involve the application of molecular biology techniques, including transgenic technology and gene editing technology (27, 28). These techniques aim to increase the expression of anti-viral genes, interfere with the virus’s genes, regulate the antiviral immune pathway, and target BmNPV genome by using CRISPR/Cas9, etc (25, 27, 29, 30). Currently, there are two main methods to cultivate silkworm varieties resistant to NPV by biotechnology: involve enhancing gene expression through methods like overexpression of *Bmhsp19.9*, *hycu-ep32*, *Bmlipase-1*, *BmPP2A*, etc (25, 31–33); additionally, the use of viral gene RNAi has been employed to suppress BmNPV mRNA, including gene silencing *ie1* (34, 35). However, there is limited research on the effectiveness of silkworm gene knockout as a means to achieve anti-BmNPV resistance. The results of this study demonstrate that *BmSPP* knockout can effectively inhibit the proliferation of BmNPV. Furthermore, this antiviral effect primarily affected the DNA replication phase of BmNPV. *BmSPP*, a seldom-reported negatively regulated antiviral gene, exhibits a strong anti-BmNPV effect.

Traditional breeding methods often have some impact on normal growth, development, and economic traits. However, researchers continue to strive for goal of breeding silkworms with strong resistance to BmNPV, without compromising economic value, cocoons quality, and ease of propagation. In this study, the knockout of *BmSPP* exhibited increased resistance to BmNPV without affecting growth and development. Importantly, there was no significant change in the rate of cocoon layer, normal survival of the offspring, and the inserted sequences can be stably passed on to the future generations through the gene editing. These findings suggest that it is possible to breed silkworms with broad prospects for resistance against BmNPV using genetic manipulation technology that target *BmSPP*.

## Data availability statement

The original contributions presented in the study are included in the article/**Supplementary Material**. Further inquiries can be directed to the corresponding author.

## Ethics statement

The manuscript presents research on animals that do not require ethical approval for their study.

## Author contributions

YF: Data curation, Investigation, Methodology, Software, Writing – original draft, Visualization. CY: Data curation, Writing – original draft, Software. LW: Data curation, Writing – original draft, Investigation. YW: Data curation, Methodology, Writing – original draft, Formal Analysis, Resources. GS: Project administration, Writing – review & editing, Funding acquisition, Methodology. PL: Funding acquisition, Project administration, Resources, Writing – review & editing, Methodology, Conceptualization, Formal Analysis.
