# Supplementary material for: BmSPP is a virus resistance gene in Bombyx mori
Source: Front Immunol. 2024 Mar 22;15:1377270. doi: 10.3389/fimmu.2024.1377270 (PMC10995218; doi:10.3389/fimmu.2024.1377270)
Supplement: Supplementary file 1 [file DataSheet_1.docx]

# Supplementary Data

Supplementary Material should be uploaded separately on submission. Please include any supplementary data, figures and/or tables.

Supplementary material is not typeset so please ensure that all information is clearly presented, the appropriate caption is included in the file and not in the manuscript, and that the style conforms to the rest of the article.

# Supplementary Figures and Tables

## Supplementary Figures


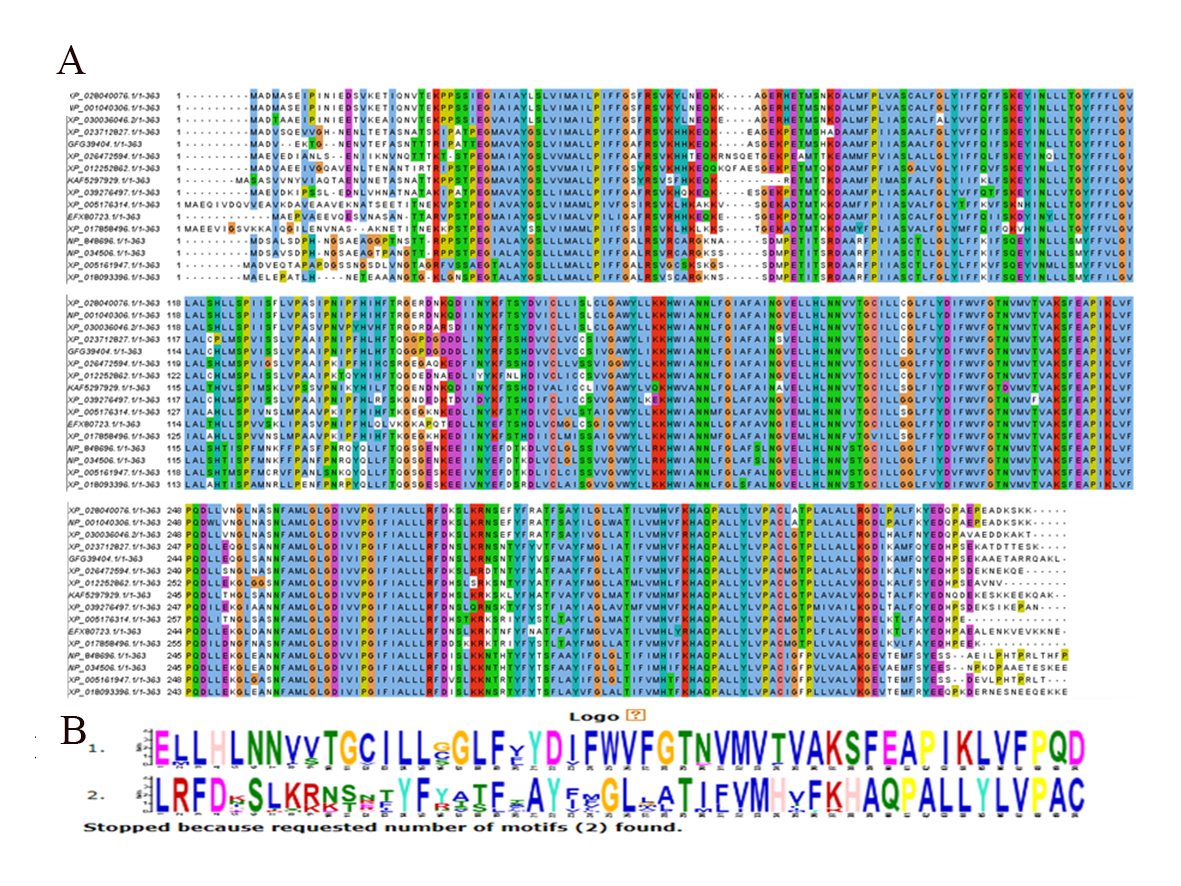


**Figure S1.** Bioinformatic analysis of *BmSPP*. **(A)** Multiple sequence alignment of *BmSPP*. **(B)** Conservative motif analysis. Amino acid sequences of *SPP* of *Bombyx mandarina* (XP_028040076.1), *Bombyx mori* (NP_001040306.1)*, Manduca sexta* (XP_030036046.2)*, Cryptotermes secundus* (XP_023712827.1)*, Coptotermes formosanus* (GFG39404.1)*, Ctenocephalides felis* (XP_026472594.1)*, Athalia rosae* (XP_012252862.1)*, Lamprigera yunnana* (KAF5297929.1)*, Nilaparvata lugens* (XP_039276497.1)*, Musca domestica* (XP_005176314.1)*, Daphnia pulex* (EFX80723.1)*, Drosophila arizonae* (XP_017858496.1)*, Homo sapiens* (NP_848696.1)*, Mus musculus* (NP_034506.1)*, Danio rerio* (XP_005161947.1)*, Xenopus laevis* (XP_018093396.1) were downloaded from NCBI.


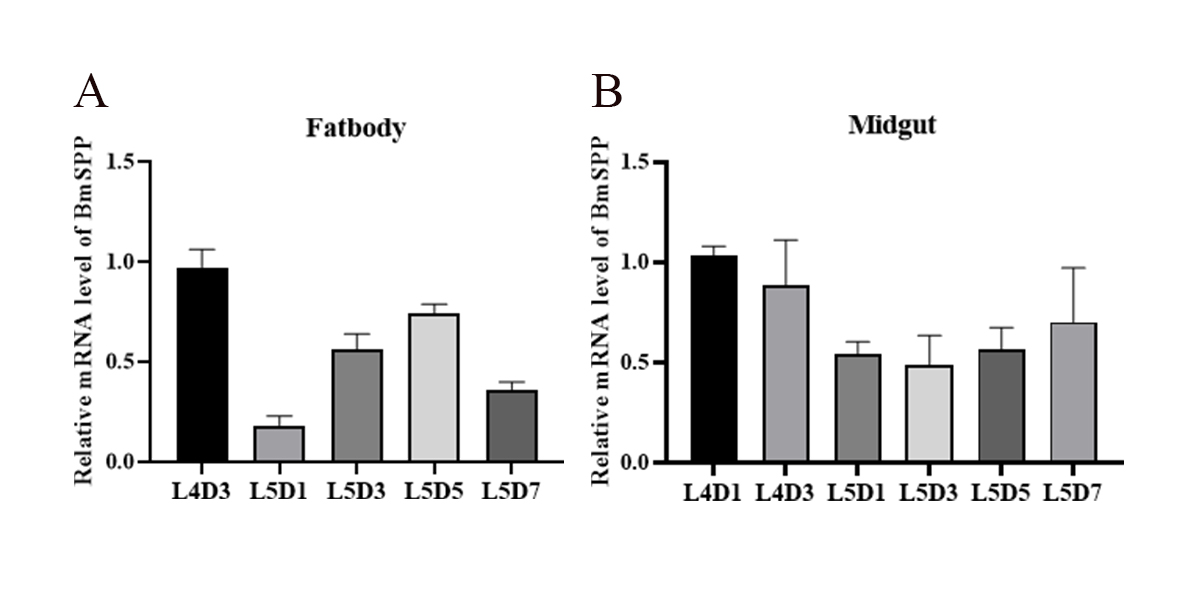


**Figure S2.** Expression of *BmSPP* in the midgut and fatbody of *Bombyx mori*, during different periods of growth. **(A)**Fatbody. **(B)** Midgut. *TIF-4A* was used as a control. Data are given as mean ±SD (n = 3).

**
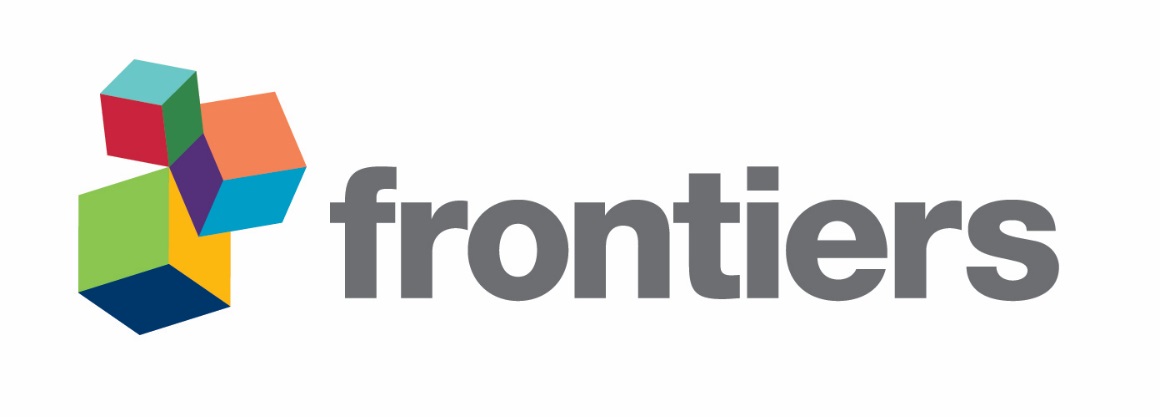
**
